# Winter Shoes

**Published:** 1878-12

**Authors:** 


### WINTER SHOES.

LIKE the gnarled oak that has withstood the storms and thunderbolts of centuries, man himself begins to die at the extremities. Keep the feet dry and warm, and we may snap our fingers in joyous triumph at disease and the doctors.

Put on two pair of thick woollen stockings, but keep this to yourself, go to some honest son of Saint Crispin and have your measure taken for a stout pair of winter boots or shoes; shoes are better for ordinary, every-day use, as they allow the ready escape of toe-odors, while they strengthen the ankles by accustoming them to depend on themselves A very slight accident is sufficient to cause a sprained ankle to an habitual boot wearer. Besides, a shoe compresses less, and hence admits of a more vigorous circulation of the blood. But wear boots when you ride or travel. Give direction, also, to have no cork or Indian rubber about the shoes, but to place between the layers of the soles, from out to out, a piece of stout hemp or tow linen which has been dipped in melted pitch. This is absolutely impervious to water—does not absorb a particle—while we know that cork does, and after a while becomes "soggy" and damp for weeks. When you put them on for the first time, with your ordinary socks, they will feel as "*easy as an old shoe*," and you may stand on damp places for hours with impunity

